# How efficient is acoustic radiation force impulse elastography for the evaluation of liver stiffness?

**Published:** 2011-07-01

**Authors:** Ioan Sporea, Radu Badea, Roxana Sirli, Monica Lupsor, Alina Popescu, Mirela Danila, Mircea Focsa, Alexandra Deleanu

**Affiliations:** 1 Department of Gastroenterology and Hepatology, University of Medicine and Pharmacy, Timisoara, Romania; 2 Ultrasonography Department, 3rd Medical Clinic, University of Medicine and Pharmacy, Cluj-Napoca, Romania; 3 Department of Biophysics and Medical Informatics, University of Medicine and Pharmacy, Timisoara, Romania

**Keywords:** Elastography, Acoustic radiation force impulse Imaging, Biopsy, Liver diseases

## Abstract

**Background:**

In chronic liver diseases, a correct estimation of the severity of liver fibrosis is important for recommendations regarding the treatment. Nowadays, evaluation of fibrosis is done by noninvasive methods such as biochemical scores and transient elastography instead of liver biopsy. The lack of sensitivity to detect fibrosis, because of its heterogeneity is a drawback of liver biopsy (LB).

**Objectives:**

To compare transient elastography (TE) and acoustic radiation force impulse (ARFI) for the evaluation of liver stiffness (LS), against percutaneous LB.

**Patients and Methods:**

Our study comprised of 223 subjects; 52 without fibrosis (38 volunteers and 14 patients with F0 on LB), 36 with F1, 40 with F2, 26 with F3 and 69 with liver cirrhosis (46 with LB and 23 with signs of cirrhosis). For each patient we performed in the same session 10 TE and 5 ARFI measurements. The median values were calculated.

**Results:**

A strong linear correlation (Spearman rho = 0.870) was found between TE and fibrosis (P < 0.0001); there was also a weaker correlation between ARFI and fibrosis (Spearman rho = 0.646; P < 0.0001). TE measurements were also correlated with ARFI measurements (Spearman rho = 0.733, P < 0.0001). The best test for predicting significant fibrosis (F ≥ 2) was TE with a cut-off value of 7.1 kPa (AUROC 0.953). For ARFI, the cut-off value was 1.27 m/s-area under ROC curve (AUROC): 0.890, sensitivity (Se) of 88.7%, specificity (Sp) of 67.5%, positive predictive value (PPV) of 64.5%, and negative predictive value (NPV) of 90% (P = 0.0044). For predicting cirrhosis (F = 4), the optimum cut-off values were 14.4 kPa for TE (AUROC: 0.985, Se: 95.6%, Sp: 94.7%, PPV: 89.2%, NPV: 98%) and 1.7 m/s for ARFI (AUROC: 0.931, Se: 93%, Sp: 86.7%, PPV: 73.6%, NPV: 96.9%) (P = 0.0102).

**Conclusions:**

LS evaluation by means of ARFI is not superior to TE for the assessment of liver fibrosis. ARFI is an accurate test for the diagnosis of cirrhosis.

## 1. Background

In chronic liver diseases a correct estimation of the severity of liver fibrosis is important for recommendations regarding the treatment (especially in chronic viral hepatitis), for prognosis and follow-up. Until a few years ago, the evaluation of fibrosis [1] was made only by means of liver biopsy (LB)-the gold standard technique for evaluation of activity and fibrosis, but later on, non-invasive methods such as biochemical scores and transient elastography (TE) emerged [2][3]. A major disadvantage of LB is its invasiveness: the risk of post-biopsy discomfort for patients and sometimes, for its serious complications [4][5][6][7]. Lack of sensitivity to detect fibrosis, because of its heterogeneity [8], is also a drawback for LB. A biopsy specimen at least 15 mm long is needed [9] for a proper histological assessment. Methods of liver fibrosis evaluation using ultrasound waves include TE (FibroScan) [10][11][12], sonoelastography (real-time tissue elastography) (RT-E) [13][14][15][16][17] and acoustic radiation force impulse elastography (ARFI) [18][19][20][21]. They are noninvasive methods, well tolerated by patients, and rapid. Another advantage of ARFI and RT-E is that the technologies are incorporated into a conventional ultrasound system. However, their value is still under evaluation. Transient elastography is an ultrasound-based method. By using an ultrasound transducer probe mounted on the axis of a vibrator, the transmission of low frequency vibrations from the right intercostal space creates an elastic shear wave that propagates into the liver. A pulse-echo ultrasound acquisition is then used to detect the velocity of wave propagation. This velocity is proportional to the tissue stiffness, with faster wave progression occurring through stiffer parts. LS measurement is then performed and the result is measured in kPa [11]. The disadvantages of the method are that the device is performing only elastography, that measurements cannot be performed in patients with ascites, and that sometimes, valid measurements cannot be made for instance in patients with lack of acoustic window (i.e., in obese patients). Sono-elastography from Siemens, a very new method still under evaluation, uses a different technology. The system enables qualitative visual and/or quantitative measurements of the mechanical stiffness properties of the tissue. Virtual Touch(TM) tissue imaging application implements ARFI technology for the evaluation of deep tissues, not accessible to superficial compression elastography techniques. Using image-based localization and a proprietary implementation of ARFI technology, shear wave speed may be quantified in a precise anatomical region, focused on a region of interest, with a predefined size, provided by the system. Measurement value and depth are also reported; the results of the elasticity are reported in m/s. The advantages of the method are that it is incorporated in an ultrasound machine, that the operator can choose the place of measurement under direct ultrasound guidance, and that the examination can be also performed in patients with ascites.

## 2. Objectives

The aim of our study was to compare two noninvasive methods for the evaluation of liver fibrosis: TE and ARFI, in patients with diffuse chronic liver diseases.

## 3. Patients and Methods

Our study was performed in Timisoara and Cluj. It included 223 patients-38 healthy volunteers, 162 patients with chronic liver diseases confirmed by LB and 23 with clinical, ultrasonographic and/or endoscopic signs of cirrhosis. The healthy volunteers were medical students, nurses and physicians from our hospital. None of them had a history of liver disease (acute or chronic). We did not perform additional tests in this subgroup (such as biological tests, viral hepatitis markers, abdominal ultrasound). However, we performed abdominal ultrasound in all patients included in the study, just before the elastographic measurements and noted the presence of liver steatosis and splenomegaly. None of the healthy volunteers had steatosis or splenomegaly. In the subgroup of 23 cirrhotic patients, the diagnosis was made based on clinical criteria, ultrasound, endoscopy, and LB. None had ascites at the moment of evaluation. They were all considered as F4 in the Metavir scoring system and they were all Child-Pugh A or B. We excluded patients with liver cirrhosis and ascites due to the fact that even if ARFI can be performed in patients with ascites, TE is not feasible in this group of patients. Liver stiffness (LS) was determined in each patient by TE (FibroScan®, EchoSens) and ARFI (Siemens Acuson S2000(TM) ultrasound system) in the same session. The LB should had been performed within six months before entering the study; none of the patients had received antiviral therapy. All the patients agreed to participate in this study. The study protocol was approved by the local Ethics Committee.

### 3.1. Transient elastography

TE was performed in all patients with a FibroScan® device (Echosens®, Paris, France) by experienced physicians (more than 1,000 examinations each). In each patient, 10 valid measurements were made; then, median of LS was calculated and reported in kPa. Only patients in whom LS measurements had a success rate (SR) of at least 60% and with an interquartile range (IQR) of less than 30% were included in our study.

### 3.2. Acoustic radiation force impulse elastography

This new type of probe automatically generates a pressure wave that propagates into the liver. Its speed, measured in m/s, is displayed on the screen. The propagation speed increases with fibrosis. The operator can select the depth at which the liver elasticity is evaluated, by placing a "measuring box" (10 mm long and 5 mm wide) in the desired place. In all our patients, Virtual Touch tissue quantification was performed, using the new Siemens Acuson S2000. The patients were examined in left lateral decubitus position with the right arm in maximum abduction. Scanning was performed between the ribs in the right liver lobe (to mask cardiac motion), with minimal scan pressure applied by the operator, while the patients were asked to stop breathing for a moment to minimize breathing motion. We performed five valid measurements in every patient; a median value was calculated; the result was reported in m/s. In 111 patients from Timisoara, ARFI measurements were made in three points: sub-capsular (0-1 cm), 1-2 cm and 2-3 cm under the capsule, and a median value was calculated and reported in m/s, trying to find out the best point to measure liver elasticity. We were able to perform measurements in the sub-capsular area in all 111 patients, but only in 95.5% (106 cases) of them at 1-2 cm below the capsule and in 85.6% (95 cases) of them at 2-3 cm below the capsule.

### 3.3. Liver biopsy

LB was performed in 112 patients using the TruCut technique with a 14G (1.8 mm in diameter) automatic needle device, Biopty Gun (Bard GMBh). Fifty patients underwent echo-assisted LB using Menghini type modified needles, 1.4 and 1.6 mm in diameter. Only LB fragments including at least six portal tracts were considered adequate for pathological interpretation and included in our study. The LBs were assessed according to the Metavir score by two senior pathologists. Fibrosis was staged on a 0-4 scale according to the Metavir score [22][23]: F0: no fibrosis; F1: portal fibrosis without septa; F2: portal fibrosis and few septa extending into lobules; F3: numerous septa extending to adjacent portal tracts or terminal hepatic venules and F4: cirrhosis. All the LBs were performed in patients with HCV chronic hepatitis for the accurate staging and grading of the liver disease.

### 3.4. Statistical analysis

Data were entered into a Microsoft Excel sheet. The analyses were done by SPSS. All the predictors for the stage of fibrosis (TE and ARFI measurements) were numeric variables, so the mean and standard deviation were calculated. Associations between assay results and fibrosis stage according to the Metavir scoring system (range: 0-4, ordinal scale) were described using the Spearman rank correlation coefficient (ρ). The diagnostic performances of the noninvasive tests were assessed by receiver operating characteristics (ROC) curves. ROC curves were built for the detection of: significant fibrosis (F ≥ 2 Metavir) and cirrhosis (F ≥ 4). Optimal cut-off values were chosen to maximize the sum of sensitivity (Se) and specificity (Sp). Se and Sp were calculated according to standard methods. Exact confidence intervals (CI) of 95% were calculated for each predictive test and used for comparing area under ROC (AUROC) curves.

## 4. Results

### 4.1. Patients

We studied 223 patients (90 men and 133 women with a mean ± SD age of 48 ± 13.05 years). The study sample included 52 subjects (23.3%) without fibrosis (38 healthy volunteers-considered F0 Metavir and 14 subjects with F0 on LB), 36 (16.1%) with F1, 40 (17.9%) with F2, 26 (11.7%) with F3 (all patients underwent LB) and 69 (30.9%) patients with liver cirrhosis (46 with LB and 23 with clinical, ultrasonographic and/or endoscopic signs of cirrhosis). The etiologies of cirrhosis in 69 patients were HCV infection in 59 (85.5%) cases, HBV infection in 2 (2.9%), alcohol abuse in 4 (5.8%) and primary biliary cirrhosis in another 4 (5.8%) cases.

### 4.2. Histological fibrosis stage

From 162 patients for whom LB was performed, 14 (8.6%) had no fibrosis (F0), 36 (22.2%) had mild fibrosis (F1), 40 (24.7%) had significant fibrosis (F2), 26 (16%) had severe fibrosis (F3), and 46 (28.4%) had cirrhosis (F4), according to the Metavir scoring system.

### 4.3. Liver stiffness measurements

**Figure 1:**
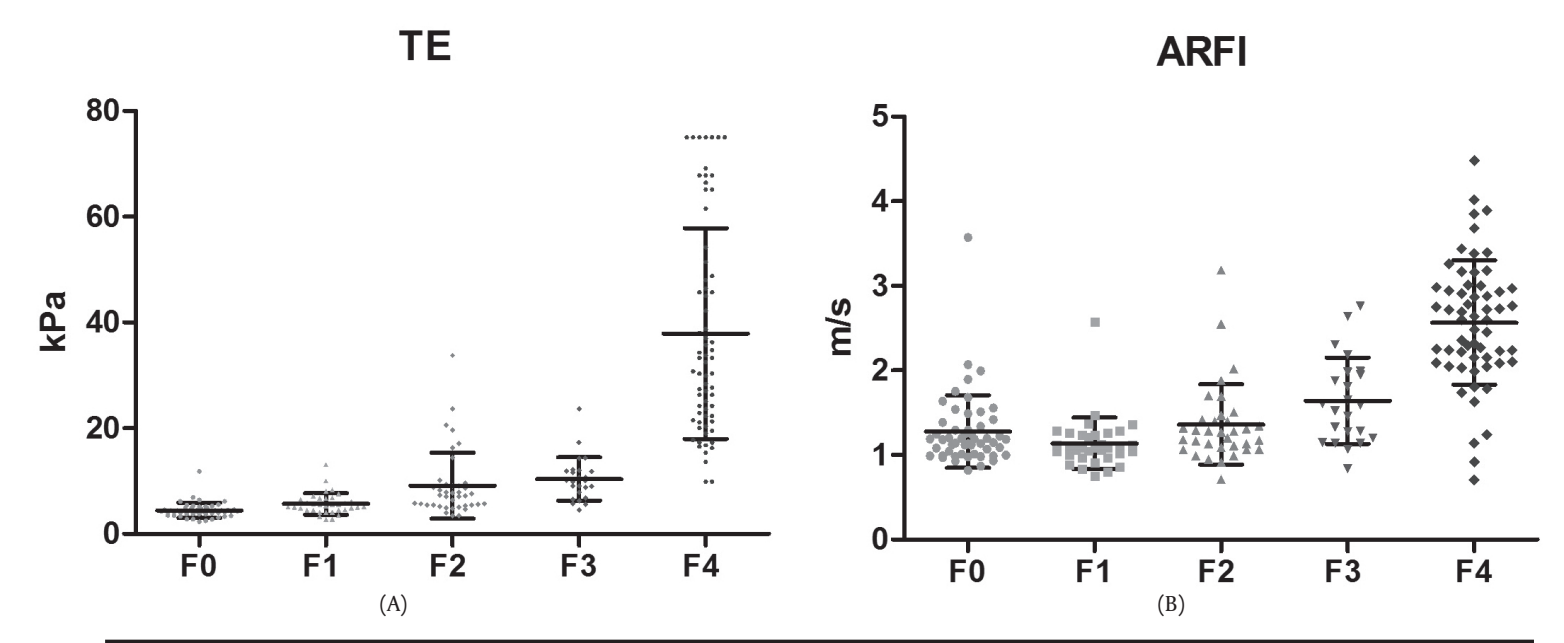
The mean value of liver elasticity assessed by TE (A) and ARFI (B)

**Figure 2:**
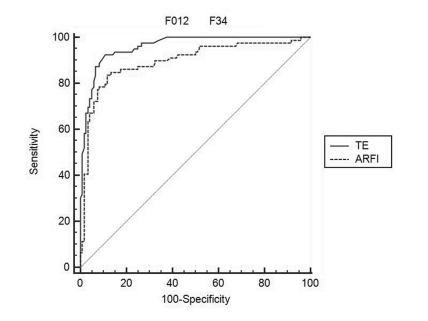
Comparative predictive values of LS measurements by TE and ARFI for prediction of significant fibrosis (F ≥ 2 Metavir)

**Figure 3:**
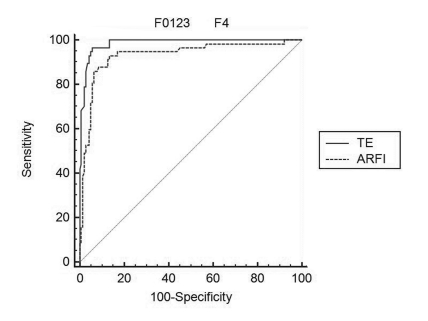
Comparative predictive values of LS measurements by TE and ARFI for prediction of cirrhosis (F = 4 Metavir).

From 223 subjects, valid LS measurements were made in 221 by TE, in 200 by ARFI and in 199 patients with both methods (there were invalid measurements in 10.8% of patients). LS measurements ranged from 2.3 to 75 kPa with TE and from 0.71 to 4.48 m/s with ARFI. A strong linear correlation (ρ = 0.870) was found between TE and fibrosis (P < 0.0001). A weaker correlation was found between ARFI and fibrosis (ρ = 0.646; P < 0.0001). TE measurements were also correlated with ARFI measurements (ρ = 0.733; P < 0.0001). The mean LS measurements according to the severity of fibrosis, assessed by TE and ARFI, are presented in Table 1 and Figure 1a and Figure 1b. The best test for predicting significant fibrosis (F ≥ 2 Metavir) was TE with a cut-off value of 7.1 kPa (AUROC of 0.953, with a Se of 93.6%, Sp of 78.7%, positive predictive value (PPV) of 76.5% and negative predictive value (NPV) of 94.3%). For ARFI, the cut-off value was 1.27 m/s (AUROC 0.890), with a Se of 88.7%, Sp of 67.5%, PPV of 64.5% and NPV of 90% (P = 0.0044) (Table 2; Figure 2). For predicting cirrhosis (F = 4 Metavir), the optimum cut-off values were 14.4 kPa for TE (AUROC = 0.985, with a Se of 95.6%, Sp of 94.7 %, PPV of 89.2% and NPV of 98%) and 1.7 m/s for ARFI (AUROC = 0.931, with a Se of 93%, Sp of 86.7%, PPV of 73.6% and NPV of 96.9%) (P = 0.0102) (Table 2; Figure 3). Comparing the results in connection with the depth of ARFI measurements (111 subjects from Timisoara), a significant, direct correlation was found between ARFI (median value of 5 measurements made 1-2 cm and 2-3 cm below the liver capsule) and the severity of liver fibrosis (ρ = 0.675 and ρ = 0.714, respectively) (P < 0.001). The subcapsular measured values of ARFI showed a poor correlation with fibrosis (ρ = 0.469). For ARFI, measurements made 1-2 cm and 2-3 cm below the liver capsule had the best predictive value for predicting significant fibrosis (F ≥ 2 Metavir), with AUROCs not significantly different from each other (0.767 and 0.731, respectively, P = 0.264). For ARFI in connection with the depth of examination, the cut-off value was 1.4 m/s for measurements made 1-2 cm below the capsule, with a Se of 71% and Sp of 78% (AUROC = 0.767); the cut-off value was 1.26 m/s for measurements made 2-3 cm below the capsule, with a Se of 75% and Sp of 64% (AUROC = 0.731).

**Table 1:** The mean value of liver elasticity assessed by TE and ARFI

**Fibrosis**	**ARFI , **m/s, No. (mean ± SD)	**TE , **kPa, No. (mean ± SD)
**0**	52 (1.28 ± 0.43)	52 (4.46 ± 1.41)
**1**	34 (1.14 ± 0.3)	35 (5.68 ± 2.04)
**2**	34 (1.36 ± 0.47)	40 (9.11 ± 6.23)
**3**	23 (1.64 ± 0.51)	25 (10.39 ± 4.1)
**4**	57 (2.60 ± 0.70)	69 (37.88 ± 19.94)

**Table 2:** Comparative predictive values of LS measurements by TE and ARFI for the prediction of significant fibrosis (F ≥ 2 Metavir) and cirrhosis (F = 4 Metavir)

	**Cut-off value**	**Se a, **%	**Sp b, **%	**PPV c, **%	**NPV d, **%	**LR+**	**LR–**	**Accuracy **%	**AUROC e**	**Std. Error**	**Asymptotic 95**%** CI**		**P value**
											**Lower Bound**	**Upper Bound**	
**Significant fibrosis**													0.0044
**ARFI f**	1.27, m/s	88.7	67.5	64.5	90	2.73	0.17	76	0.890	0.0254	0.844	0.934	
**TE g**	7.1, kPa	93.6	78.7	76.5	94.3	4.4	0.08	86.4	0.953	0.0122	0.922	0.982	
**Cirrhosis**													0.0102
**ARFI**	1.7, m/s	93	86.7	73.6	96.9	7	0.08	88	0.931	0.0227	0.886	0.962	
**TE**	14.4, kPa	95.6	94.7	89.2	98	18.2	0.05	94.1	0.985	0.0064	0.957	0.997	

^a^ SE: Sensivity

^b^ SP: Specificity

^c^ PPV: Positive predictive value

^d^ NPV: Negative predictive value

^e^ AUROC: Area under ROC curve

^f^ ARFI: Acoustic radiation force impulse

^g^ TE: Transient elastography

For predicting cirrhosis (F = 4 Metavir), the optimum cut-off values were 1.8 m/s for measurements made 1-2 cm under the capsule (AUROC = 0.929) and 1.78 m/s for measurements made 2-3 cm under the capsule (AUROC = 0.951).

## 5. Discussion

The correct evaluation of liver fibrosis in chronic diffuse hepatopathies is of paramount importance for the management of these diseases. LB is still considered the gold standard for the assessment of severity of fibrosis. Studies comparing the noninvasive methods of evaluation in chronic liver disease with LB have been conducted to assess whether they merit replacing this invasive method in the future. Considering the fact that fibrosis is heterogeneously distributed in the liver, LB has been criticized in the past because it evaluates only 1/50,000 of the total volume of the liver, due to the small volume of the tissue sample [7]. It has been shown that liver fragments obtained in the same session by laparoscopic biopsy from the left and right liver lobes revealed different stages of fibrosis in almost half of the patients [8]. In 14.5% of the cases, cirrhosis was present in one of the lobes but not in the other and in 33.1% of the cases the stage of fibrosis was higher in one of the lobes by at least one point [8]. By means of percutaneous LB, tissue samples 1-4 cm in length are obtained (preferably at least 1.5 cm) whatever the kind of needle used [4]. Also, one must consider that the smaller the liver sample size is, the higher is the chance to subevaluate the severity of the liver disease [24][25]. Using a mathematical model, Bedossa [9] estimated that the chance of misdiagnosis in a fragment 2.5 cm in length can be as high as 25% and that the optimal size of a LB sample is 4 cm (difficult enough to obtain in daily practice). Also we must not forget that LB is an invasive method which would cause anxiety to the patient who has to undergo the procedure and that LB is not totally risk-free. Published data state that serious complications following diagnostic LB may occur in 1%-5% of the cases [5][6] and, also, that the death rate following diagnostic LB can reach 1-3/10,000 of the biopsied cases [4][7]. On the other hand, TE assessment of LS was validated as a method of evaluation in chronic HCV hepatitis. Furthermore, there are some papers that proved the value of this method in other chronic hepatopathies (such as HBV chronic infection, hemochromatosis, primary billiary cirrhosis or non-alcoholic steatohepatitis) [26][27][28][29][30][31]. Two meta-analyses [11][27] demonstrated that this method is very good for the diagnosis of cirrhosis and advanced fibrosis. Even if our study may have a possible error factor, due to the fact that it included not only patients who had undergone LB (considered to be the "gold standard" for hepathological evaluation), but also patients who did not have any known hepatic pathology (considered to be "normal"), as well as patients with known liver cirrhosis (some without morphologic exam), it seems that TE can be considered a reliable diagnostic modality for clinical practice. In our study, the optimum LS cut-off values for liver cirrhosis (F = 4 Metavir) were 14.4 kPa for TE (AUROC = 0.985, with a Se of 95.6%, Sp of 94.7 %, PPV of 89.2% and NPV of 98%) and 1.7 m/s for ARFI (AUROC = 0.931, with a Se of 93%, Sp of 86.7%, PPV of 73.6% and NPV of 96.9%), TE had a better predictive value (P = 0.0102). The best test for predicting significant fibrosis (F ≥ 2 Metavir) was again TE, with a cut-off value of 7.1 kPa (AUROC = 0.953, with a Se of 93.6%, Sp of 78.7%, PPV of 76.5% and NPV of 94.3%). For ARFI, the cut-off value was 1.27 m/s (AUROC = 0.890, with a Se of 88.7%, Sp of 67.5%, PPV of 64.5% and NPV of 90%) (P = 0.0044). ARFI elastography is a new technology available on the Siemens Acuson S2000 ultrasound system and its use is not well established yet. In a previous study performed by our group [21] ARFI was not superior to TE for the assessment of liver fibrosis but was an accurate test for the diagnosis of cirrhosis. The present study aimed at validating the previous results on a larger group of patients. ARFI is a new method, still under evaluation so we tried to find the ideal place to measure liver elasticity (it is not specified by the producer). In all 111 subjects from the Timişoara subgroup, we performed measurements in 3 points: subcapsular (0-1 cm below the liver capsule), at 1-2 cm under the capsule and at 2-3 cm under the capsule. We were able to perform measurements in the subcapsular area in all 111 subjects who were evaluated by ARFI, but only in 95.5% (106 cases) of them at 1-2 cm below the capsule and in 85.6% (95 cases) of them at 2-3 cm below the capsule. The best correlation with fibrosis was obtained for measurements made at 2-3 cm below the capsule (ρ = 0.714) and at 1-2 cm under the capsule (ρ = 0.675). Considering all these facts, probably the best place for ARFI determinations should be 1-2 cm below the capsule. In our study we found a linear correlation between ARFI and fibrosis (ρ = 0.646; P < 0.0001). TE measurements were also correlated with ARFI measurements (ρ = 0.733, P < 0.0001). For ARFI, the cut-off value for predicting significant fibrosis (F ≥ 2 Metavir) was 1.27 m/s (AUROC = 0.890), with a Se of 88.7%, Sp of 67.5%, PPV of 64.5% and NPV of 90% (P = 0.0044). For predicting cirrhosis (F = 4 Metavir), the optimal cut-off value was 1.7 m/s for ARFI (AUROC = 0.931, with a Se of 93%, Sp of 86.7%, PPV of 73.6% and NPV of 96.9%) (P = 0.0102). Several other studies evaluated the performance of this method [18][19][20][32][33][34][35]. In a study performed by Friedrich-Rust [18], in which ARFI was compared to LB and blood markers in 86 patients with chronic hepatitis (HBV or HCV), the Spearman correlation coefficients between the histological fibrosis stage and ARFI, TE, FibroTest and APRI scores, indicated significant correlations of 0.71, 0.73, 0.66, and 0.45, respectively (P < 0.001). In the study performed by Lupşor and co-workers [19], 112 consecutive patients with chronic HCV hepatitis were evaluated through histology (Metavir score), ARFI and TE. In this study, ARFI was correlated with liver fibrosis (r = 0.717, P < 0.0001) and necroinflammatory activity (r = 0.328, P < 0.014), but not with steatosis (r=0.122, P = 0.321). In this study there was a significant increase in mean ± SD ARFI values in parallel with the increase in fibrosis stage as follows: 1.079 ± 0.150 m/s (F0-F1), 1.504 ± 0.895 m/s (F2), 1.520 ± 0.575 m/s (F3), 2.552 ± 0.782 m/s (F4) (P < 0.0001), but there was a certain degree of overlap between the consecutive stages F1-F2 (P = 0.072) and F2-F3 (P = 0.965). In this study the cut-off values predictive for each fibrosis stage were 1.19 m/s for F ≥ 1, 1.34 for F ≥ 2, 1.61 for F ≥ 3 and 2.00 m/s for F4. Concerning the comparison between ARFI and TE, this study found that the AUROCs were 0.709 vs. 0.902 (P = 0.006) for F ≥ 1; 0.851 vs. 0.941 (P = 0.022) for F ≥ 2; 0.869 vs. 0.926 (P = 0.153) for F ≥ 3; and 0.911 vs. 0.945 (P = 0.331) for F4. Fierbinţeanu-Braticevici, et al. [20] compared ARFI elastography, APRI index and FibroMax in a consecutive series of 74 patients who underwent LB for HCV chronic hepatitis and showed that the diagnostic accuracy of ARFI elastography, expressed as AUROC had a validity of 90.2% (95% CI: 83.1%-97.2%, P < 0.001) for the diagnosis of significant fibrosis (F ≥ 2). Also ARFI sonoelastography performed better for F3 or F4 fibrosis (AUROC = 0.993; 95% CI: 0.979-1). On the other hand, in the study by Takahashi, et al. [32] the AUROC curves were 0.94 (95% CI: 0.87-0.99) for F2-F4, 0.94 (95% CI: 0.88-0.99) for F3-F4 and 0.96 (95% CI: 0.91-1.01) for F4. The cut-off values of the shear wave velocity were as follows: > 1.34 m/s for F2-F4 (Se of 91.4%, Sp of 80%); > 1.44 m/s for F3-F4 (Se of 96.2%, Sp of 79.3%); and > 1.80 m/s for F4 (Se of 94.1%, Sp of 86.8%). The studies that we presented [18][19][20][32], together with the present study showed that there is a strong correlation between histological fibrosis and ARFI measurements, also that the best performances of this method are for the prediction of severe fibrosis and cirrhosis and that ARFI is not better than TE for the evaluation of liver stiffness. Therefore, the use of ARFI measurements could be an advantage, being a "real-time" evaluation of LS. It can be also used in patients in which valid measurements of LS by TE could not be obtained (since the location of ARFI measurement can be chosen under direct ultrasound guidance), and also in patients with ascites. Also, ARFI is a rapid method for the assessment of liver fibrosis, totally free of adverse events, comfortable for both the patient and the examiner (with a mean duration of approximately 5 minutes). So, immediately after an ultrasound evaluation of the liver, ARFI measurements can be done so that information regarding the severity of liver fibrosis are available on the spot, without having to buy another machine such as the FibroScan, which is quite expensive (around 80,000 Euros).

Our study demonstrates that, at the present time, LS evaluation by means of ARFI is not superior to TE (FibroScan) for the assessment of liver fibrosis. Also, there is a strong correlation between histological fibrosis and ARFI measurements. The best performance of this method was shown to be for the prediction of severe fibrosis and cirrhosis. Another advantage of ARFI is probably the fact that this system is integrated in an ultrasound machine, which already exist in some ultrasound departments.
